# Naringin attenuates acute myocardial ischemia-reperfusion injury via miR- 126/GSK-3β/β-catenin signaling pathway

**DOI:** 10.1590/acb370102

**Published:** 2022-04-08

**Authors:** Xiuhui Guo, Qinghong Ji, Mei Wu, Weihong Ma

**Affiliations:** 1 MD. Department of Price Office of Finance - The Second Hospital - Cheeloo College of Medicine - Shandong University - Shandong Province, China.; 2 MD. Department of Obstetrics - The Second Hospital - Cheeloo College of Medicine - Shandong University - Shandong Province, China.; 3 MD. Department of Ultrasonography - The Second Hospital - Cheeloo College of Medicine - Shandong University - Shandong Province, China.

**Keywords:** Reperfusion Injury, Myocardial Ischemia, Flavonoids, Rats

## Abstract

**Introduction:**

Myocardial ischemia-reperfusion (I/R) injury is one of the mechanisms contributing to the high mortality rate of acute myocardial infarction.

**Purpose:**

This study intended to study the role of naringin in cardiac I/R injury.

**Methods:**

AC16 cells (human cardiomyocyte cell line) were subjected to oxygen-glucose deprivation/recovery (OGD/R) treatment and/or naringin pretreatment. Then, the apoptosis was examined by flow cytometry and Western blotting. The concentration of IL-6, IL-8 and TNF-α was measured by enzyme-linked immunosorbent assay (ELISA) kits. How naringin influenced microRNA expression was examined by microarrays and quantitative real-time polymerase chain reaction (qRT-PCR). Dual luciferase reporter assay was employed to evaluate the interaction between miR-126 and GSK-3β. The GSK-3β/β-catenin signaling pathway was examined by Western blotting. Finally, rat myocardial I/R model was created to examine the effects of naringin *in vivo*.

**Results:**

Naringin pretreatment significantly decreased the cytokine release and apoptosis of cardiomyocytes exposed to OGD/R. Bioinformatical analysis revealed that naringin upregulated miR-126 expression considerably. Also, it was found that miR-126 can bind GSK-3β and downregulate its expression, suggesting that naringin could decrease GSK-3β activity. Next, we discovered that naringin increased β-catenin activity in cardiomyocytes treated with OGD/R by inhibiting GSK-3β expression. Our animal experiments showed that naringin pre-treatment or miR-126 agomir alleviated myocardial I/R.

**Conclusions:**

Naringin preconditioning can reduce myocardial I/R injury via regulating miR-126/GSK-3β/β-catenin signaling pathway, and this chemical can be used to treat acute myocardial infarction.

## Introduction

It has been estimated that nearly ischemic heart disease (IHD) has affected 1.72% of the total world population, and its incidence worldwide keeps increasing^1^. From a pathological perspective, IHD is mainly caused by the reduced blood supply to heart. Therefore, two revascularization strategies–percutaneous coronary intervention (PCI) and coronary artery bypass grafting (CABG)–have been invented^2^. Yet, these two revascularization methods carry the risk of cardiac ischemia/reperfusion (I/R) injury. During I/R, there are increased oxidative stress, inflammatory response, and impaired autophagy^3^. To minimize heart damage, many treatment strategies have been created, for example, suppressing immune response, and decreasing high-blood pressure^4^. Despite these advances, the mortality of IHD remains very high, which requires novel treatments.

Naringin, which is a natural flavanone glycoside, is a main active chemical component from Chinese herbs such as *Drynaria fortunei* and *Citrus aurantium* 5. It has been proven to promote bone generation, suppress inflammation, inhibit cancer growth, attenuate oxidative stress, regulate cell metabolism, and improve neurodegenerative diseases^6^^,^ 7. Also, naringin shows protective effects on cardiovascular diseases. For example, it can attenuate the toxic effects of doxorubicin and bisphenol on cardiomyocytes via regulating reactive oxygen species (ROS) level and p38/MAPK signaling pathway^8^^,^ 9. Another study showed that naringin could reduce cardiac inflammatory response^10^. Research in other fields has indicated that naringin can attenuate intestinal, testicular, and cerebral I/R injury^11^^-^ 13. Yet, its role in cardiac I/R injury remains unknown. Therefore, this research aimed to investigate if naringin could exert any protective effects on cardiac I/R injury.

In this research, we hypothesized that naringin could attenuate cardiac I/R-induced injury. AC16 cells (human cardiomyocytes) were exposed to oxygen-glucose deprivation/recovery (OGD/R) treatment, and naringin was employed to pre-treat AC16 cells. In addition, rat I/R model was constructed to validate our findings *in vitro*. The following is what we have discovered:

- naringin ameliorates OGD/R-induced apoptosis and inflammation of cardiomyocytes;
- naringin can modulate GSK-3β/β-catenin signaling pathway via miR-126;
- miR-126 can bind GSK-3β;
- naringin can alleviate cardiac I/R injury *in vivo*.

These findings suggest that naringin has therapeutic potential to minimize cardiac I/R injury.

## Methods

### Cells and cell culture

AC16 cells (human cardiomyocytes) were bought from the cell bank of the Institute of Biochemistry and Cell Biology of the Chinese Academy of Sciences (Shanghai, China). These cells were cultured in Dulbecco’s modified Eagle’s medium (DMEM) (Sigma-Aldrich, United States of America) with 10% fetal bovine serum (Gibco, United States of America). AC16 cells were incubated at 37°C in a humid atmosphere with 5% CO_2_ and would be passaged or used for further experiments when the confluence reached 70-80%.

### Cardiomyocyte oxygen-glucose deprivation/recovery model construction

Our OGD/R model was established as previously described^15^. AC16 cells were initially cultured in DMEM at 37°C and 20% O_2_. Then, they were washed with phosphate buffer saline (PBS) and incubated with glucose-free and serum-free DMEM medium at 37°C under 2% O_2_ for 6 h to induce *in-vitro* ischemia. After that, these cells were then cultured in DMEM with 10% FBS at 37°C under 20% O_2_ for 6 h to simulate reperfusion. The control group was always cultured in normal conditions (37°C and 20% O_2_). To test the effects of naringin on AC16 cells, they were pretreated with naringin and then underwent the induction of ischemia and reperfusion. Naringin was bought from MedChemExpress (United States of America) (Cat. No: HY-N0153), and its purity was more than 98% according to the manufacturer’s instructions.

### Cell transfection

MiR-129 mimic, mimic negative control (NC), miR-129 inhibitor and inhibitor negative control (NC) were compounded by Genomeditch (Shanghai, China). These oligonucleotides were transfected into AC16 cells at the concentration of 40 nm/L, based on the manufacture’s protocol. Lipofectamine 3000 (Invitrogen, United States of America) was employed as a transfection reagent.

### Quantitative real-time polymerase chain reaction

Trizol (Sigma-Aldrich, United States of America) was employed to obtain total RNA, and miRNA was obtained by using Molpure Cell/Tissue miRNA kit (Yeasen, Shanghai, China). In addition, mRNA was transcribed via Hifair III One Step quantitative real-time polymerase chain reaction (RT-qPCR) probe kit (Yeasen, Shanghai, China), and TaqMan MicroRNA reverse transcription kit (Invitrogen, United States of America) was used to transcribe miRNA. SYBR green was from Roche (Switzerland). U6 was the endogenous control for miRNAs, and GAPDH was the endogenous control for mRNA (Table 1).

**Table 1:** The primers of the study.

Gene	Species	Primer 5’→3’
MiR-126	Human	F: GCTGGCGACGGGACATTAT
		R: CGGCGCATTATTACTCACGG
U6	Human	F: GAACTGCTTCATTCGGGGCT
		R: TGGGACTTGGGGTTATGGGT
IL6	Rat	F: CTGCAAGAGACTTCCATCCAG
		R: AGTGGTATAGACAGGTCTGTTGG
IL8	Rat	F: ATGCCCTCTATTCTGCCAGAT
		R: GGTGCTCCGGTTGTATAAGATGA
GAPDH	Rat	F: TGACCTCAACTACATGGTCTACA
		R: CTTCCCATTCTCGGCCTTG

CCK-8 assay

AC16 cells (40,000-60,000 cells per well) were placed onto 96-well plates and exposed to different concentration of naringin afterwards CCK-8 solution (Beyotime, China) was mixed with samples. Next, these cells would be cultured at room temperature for 10 min. After that, cell viability was measured according to manufacturer’s instruction.

### Enzyme-linked immunosorbent assay kits

Enzyme-linked immunosorbent assay (ELISA) kits for detecting the concentration of IL-6, IL-8 and TNF-α were bought from Abcam (United Kingdom), and their respective catalogue numbers were ab178013, ab214030, and ab181421. Cytokines were extracted from the supernatant following the protocols provided by Abcam. After that, ELISA kits were employed to detect the concentration of cytokines.

### Dual luciferase reporter activity assay

Initially, AC16 cells were plated onto 96-well plates. When their confluence grew to 60-70%, AC16 cells were co-transfected with pGL3-GSK3B-WT or pGL3-GSK3B-MUT and miR-129 mimic NC, miR-129 mimics, miR-129 inhibitor NC or miR-129inhibitor, respectively. At 48 h after the transfection, the luciferase activity of these samples was examined according to Promega’s instructions.

### Western blotting

Cell lysates were obtained via utilizing radioimmunoprecipitation assay (RIPA) buffer (Beyotime, Shanghai, China) added with protease inhibitor cocktail (Beyotime, Shanghai, China). Proteins were loaded into SDS-PAGE gel. After electrophoresis, proteins were transferred onto polyvinylidene fluoride membrane (Thermo Fischer Scientific, United States of America). These membranes were blocked with 4% bovine serum albumin for 1 h at room temperature, after which they were incubated with primary antibodies at 4°C for about 24 h. Next, these membranes were incubated with the appropriate secondary antibodies at room temperature for around 1 h. Finally, protein bands would be visualized by excellent chemiluminescent substrate (ECL) kits (Millipore, United States of America). The primary antibodies against GSK-3β (1:1,000, Abcam, United Kingdom; ab32291), β-catenin (1:1,000, Abcam, United Kingdom; ab32572), cleaved caspase-3 (1:1,000, Abcam, United Kingdom; ab32042), BAX (1:1,000, Abcam, United Kingdom; ab32503), PCNA (1:1,000, Abcam, United Kingdom; ab92552), and GAPDH (1:1,000, Beyotime, China; AF1186) were used in this research. GAPDH was used as the internal control.

### Flow cytometry

In brief, FITC annexin V and PI (Thermo Fischer Scientific, United States of America) were added into each sample to stain cells. What came next was the incubation of these cells at about 24°C for 10 min and the stained cells would be analyzed. The data was processed by FlowJo (Three Star, United States of America).

### Rat I/R model

Our animal experiments were approved by the Ethical Committee of the Second Hospital, Cheeloo College of Medicine, Shandong University, and they were performed under the guidelines published by the committee.

Twenty-eight Sprague-Dawley rat (3 months old, male, 200 g) were bought from our animal center and kept at animal rooms (10-h light/14-h dark cycle, 22-27°C, relative humidity: 40-60%) under sterile environment. These animals were randomly divided into four groups:

- The control group (n = 7);
- The I/R group (n = 7);
- The I/R + naringin co-treatment group (n = 7);
- The I/R + miR-129 agomir co-treatment group (agomirs were purchased from Genomeditch, Shanghai).

In order to induce myocardial infarction, rats were initially anesthetized with ketamine (100 mg/kg i.p.). Two h before the induction of AMI, rats were intraperitoneally administered with naringin (50 mg/kg) or miR-129 agomir (50 mg/kg). Then, the left anterior descending coronary artery was exposed, and it was ligated with nylon sutures for 30 min. After that, the ligation was withdrawn to allow blood reperfusion, and rat’s chest wall was closed. After the procedure, rats were transferred to the animal facility for recovery. The hearts were collected on Days 1, 2, 3, 4, 5, 6, and 7. Rats were sacrificed by cervical dislocation, and tibia was collected for further research.

### Hematoxylin-eosin staining and TUNEL staining

In brief, 4% paraformaldehyde was used for fixing mouse heart tissue for 24 h. The hippocampus was embedded in paraffin, and 5-μm thick sections were obtained. Following hematoxylin-eosin (H&E) staining, tissue sections were observed under a microscope. Apoptosis was measured using a TUNEL assay kit (Invitrogen, United States of America). Images were analyzed by Image-Pro Plus 6.

### Statistical methods

Our data was analyzed by operating on GraphPad Prism 8.0 and illustrated as mean ± standard deviation (SD). All our experiments were performed five times independently. Student’s t-test and one-way analysis of variance (ANOVA, Bonferroni post hoc test) were adopted in our analysis, depending on the experiment. Two-tailed P < 0.05 was considered as carrying statistical significance.

## Results

### Naringin reduces OGD/R-induced apoptosis and inflammation of cardiomyocytes

**Figure 1:**
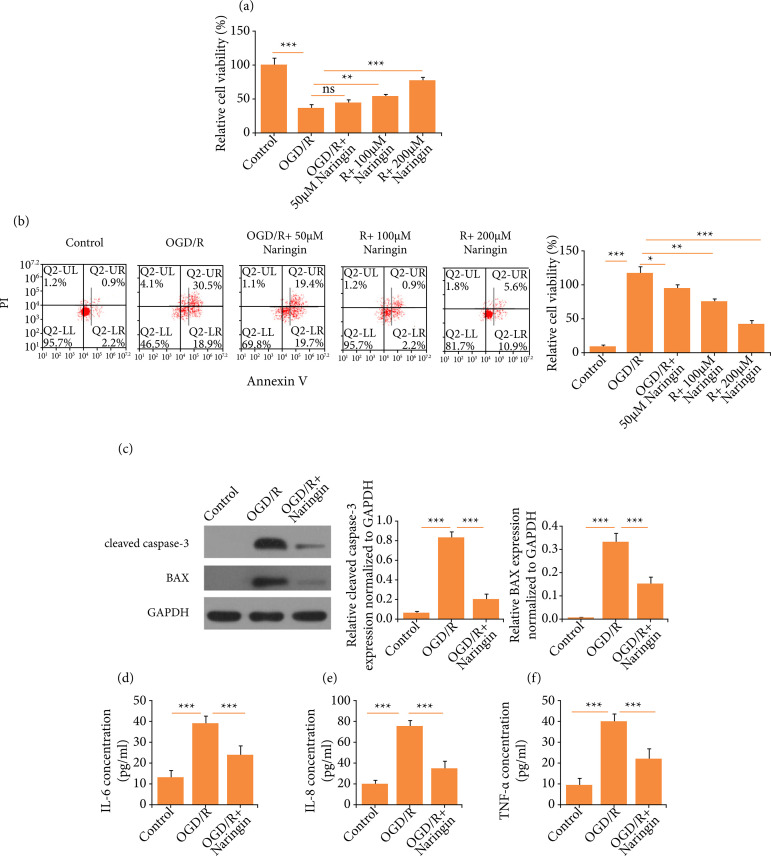
Naringin reduces oxygen-glucose deprivation/recovery (OGD/R)-induced apoptosis and inflammation of cardiomyocytes. **(a)** Cell viability following OGD/R and/or naringin treatment. **(b)** Flow cytometry to detect cell apoptosis. **(c)** Western blots for cleaved caspase-3 and BAX. **(d)** IL-6 concentration. **(e)** IL-8 concentration. **(f)** TNF-α concentration.

To simulate AMI *in vitro*, AC16 cells were subjected to OGD/R. To test the efficacy of naringin, various concentrations of naringin were employed to pre-treat cardiomyocytes, and then cell viability was examined by CCK-8 assay. As it is shown in Fig. 1a, naringin increased the cell viability in a dosage-dependent way. The results of flow cytometry showed that the apoptotic percentage of AC16 cells could be significantly reduced by naringin (Fig. 1b). Also, the expression of cleaved caspase-3 and BAX was decreased by naringin (Fig. 1c). Therefore, we chose 200-μM naringin for the subsequent experiments. In the meantime, we tested the effects of naringin on the release of cytokines from AC16 cells. As it is illustrated in Fig. 1 d-f, the concentration of IL-6, IL-8, and TNF-α in AC16 cells was increased significantly following OGD/R treatment, whereas the concentration of these inflammatory factors was downregulated by naringin. Given these results, we thought that the apoptosis and inflammatory response of cardiomyocytes could be attenuated by naringin.

NS: non-significant; *P < 0.05; **P < 0.01; ***P < 0.001, versus the control group.

### Naringin exerts its anti-apoptotic and anti-inflammatory effects via miR-126

**Figure 2:**
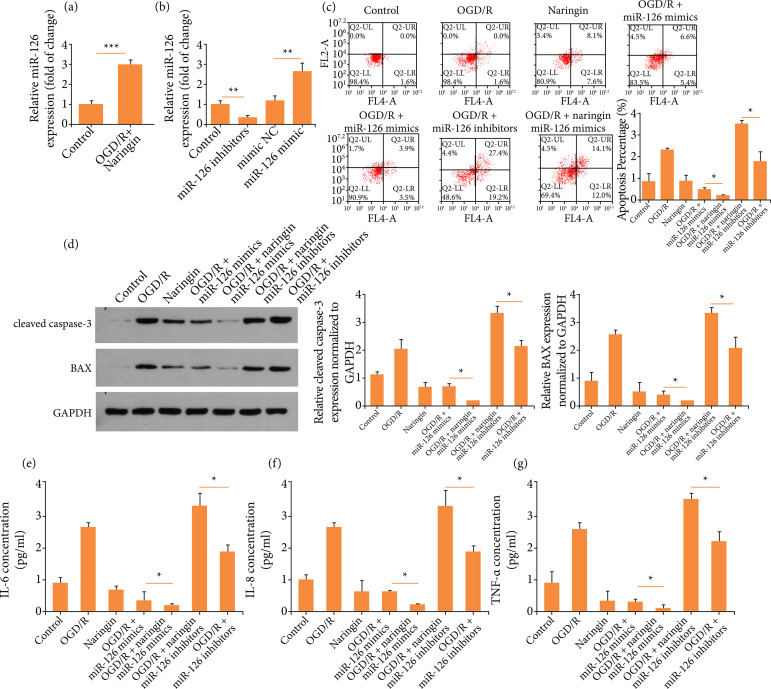
Naringin exerts its anti-apoptotic and anti-inflammatory effects via miR-126. **(a)** MiR-126 expression. **(b)** MiR-126 expression after transfection. **(c)** Flow cytometry to detect cell apoptosis. **(d)** Western blots for cleaved caspase-3 and BAX. € IL-6 concentration. **(f)** IL-8 concentration. **(g)** TNF-α concentration.

It was reported that naringin could modulate miR-126, so we hypothesized that naringin might regulate miR-126 to mediate its effects^14^. We found that the co-treatment of OGD/R and naringin increased miR-126 expression, when compared to the OGD/R treatment group (Fig. 2a). To explore the role of miR-126, the expression of miR-126 was upregulated or downregulated by transfecting AC16 cells with miR-126 mimic or miR-126 inhibitor (Fig. 2b). Following the transfection, these cells were subject to OGD/R and/ornaringin treatment. As it is displayed in Fig. 2 c-d, AC16 cells treated with OGD/R and miR-126 mimic transfection exhibited less apoptosis, in contrast to those exposed to OGD/R. Moreover, inhibiting miR-126 in AC16 cells which were treated with OGD/R resulted in to exaggerate apoptosis. Next, we found that AC16 cells treated with OGD/R and miR-126 mimic transfection exhibited reduced generation of IL-6, IL-8 and TNF-α, whereas downregulating miR-126 could promote the inflammatory response of AC16 cells (Fig. 2 e-g). These data suggest that miR-126 might mediate the anti-apoptotic and anti-inflammatory effects of naringin.

NS: non-significant; *P < 0.05; **P < 0.01; ***P < 0.001, *vs*. the control group.

### MiR-126 can target GSK3B

**Figure 3:**
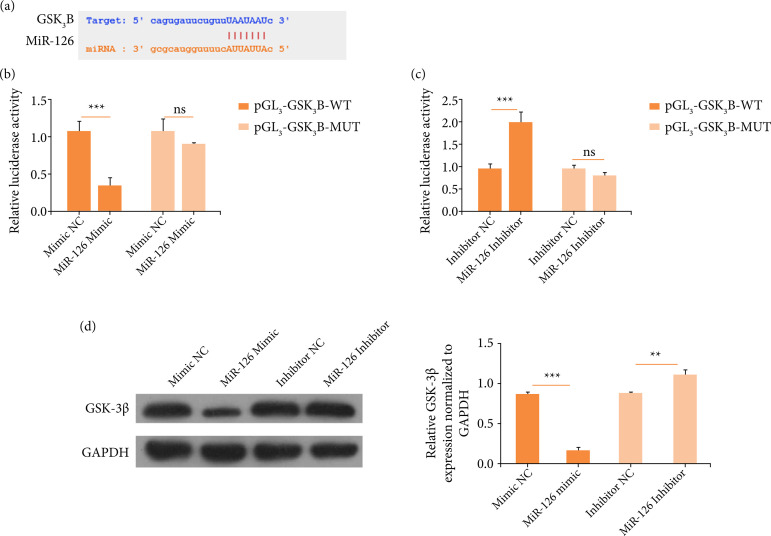
MiR-126 can target GSK3B. **(a)** The complementary sequence between miR-126 and GSK3B. **(b)** Relative luciferase activity. **(c)** Relative luciferase activity. **(d)** Western blots for GSK-3β expression.

To investigate the downstream effector of miR-126, we used StarBase, which could predict the binding sequence between miRNAs and genes. It was predicted that GSK3B could be bound by miR-126 (Fig. 3a). To confirm the interaction between miR-126 and GSK-3β, dual luciferase reporter assay was performed. AC16 cells were co-transfected with pGL3-GSK3B-WT and/or miR-126 mimic, mimic NC, miR-126 inhibitor, and inhibitor NC. AC16 cells with pGL3-GSK3B-WT and miR-126mimic transfected showed lower luciferase activity, in contrast to the control group (Fig. 3b). In addition, AC16 cells with pGL3-GSK3B-WT and miR-126 inhibitor transfected showed higher luciferase activity when compared to the control group (Fig. 3c). Moreover, it was discovered that GSK-3β expression was downregulated when AC16 cells were transfected with miR-126 mimic, but its expression was upregulated when AC16 cells were transfected with miR-126 inhibitor (Fig. 3d). These results indicate that miR-126 could directly modulate GSK-3β expression.

NS: non-significant; *P < 0.05; **P < 0.01; ***P < 0.001, *vs*. the control group.

### Naringin attenuates oxygen-glucose deprivation/recovery-induced apoptosis of cardiomyocytes via GSK-3β/β-catenin signaling pathway

**Figure 4:**
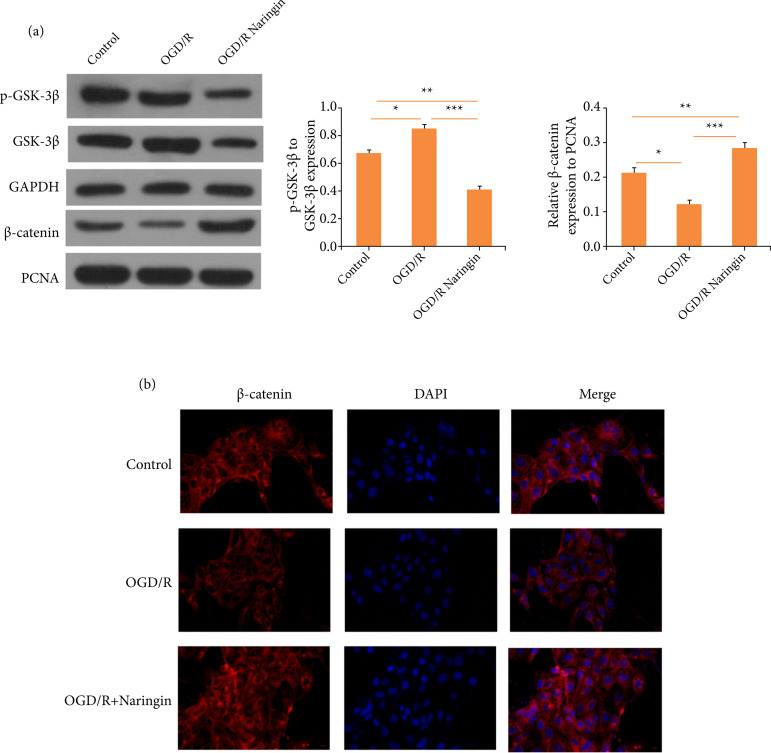
Naringin attenuates oxygen-glucose deprivation/recovery (OGD/R)-induced apoptosis of cardiomyocytes via GSK-3β/β-catenin signaling pathway. **(a)** Western blots for GSK-3β/β-catenin signaling pathway. **(b)** Immunofluorescence staining for β-catenin.

Our previous research has suggested that naringin could regulate miR-126 and that miR-126 targets GSK-3β. Therefore, we hypothesized that naringin could modulate GSK-3β/β-catenin signaling. To confirm this, Western blotting was carried out. As it is shown in Fig. 4 a-b, OGD/R treatment increased the phosphorylation of GSK-3β and more β-catenin in AC16 cells. Yet, the co-treatment of OGD/R and naringin remarkably reduced the phosphorylation of GSK-3β, and the β-catenin expression was increased. Therefore, naringin could modulate the GSK-3β/β-catenin signaling pathway in cardiomyocytes.

NS: non-significant; *P < 0.05; **P < 0.01; ***P < 0.001, *vs*. the control group.

### Naringin ameliorates myocardial ischemia-reperfusion-induced damage via miR-126/GSK-3β signaling pathway

**Figure 5:**
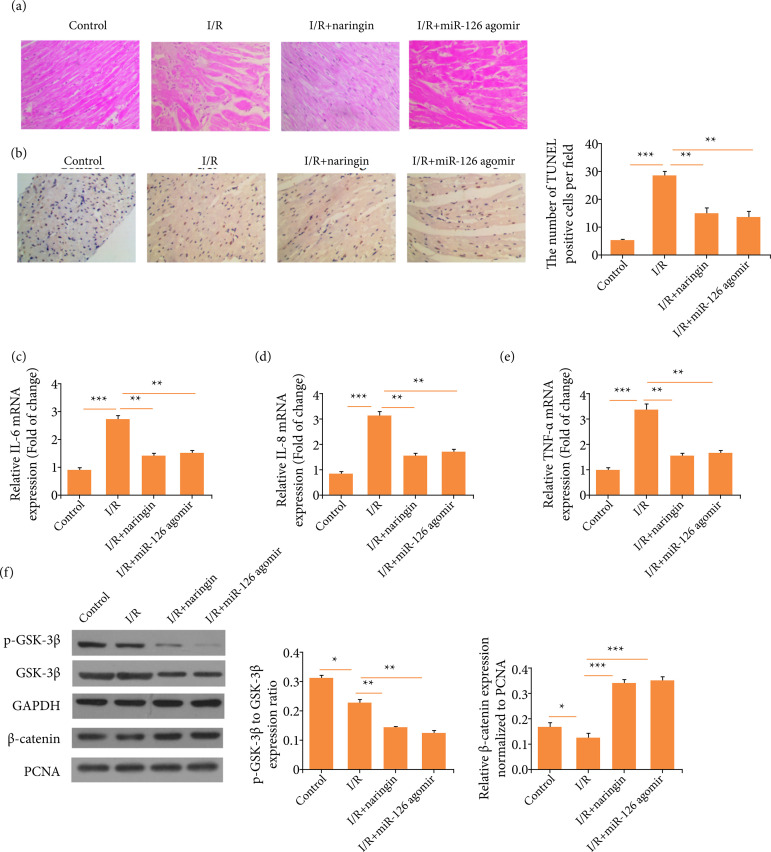
Naringin ameliorates myocardial ischemia-reperfusion (I/R)-induced damage via miR-126/GSK-3β signaling pathway. **(a)** Hematoxylin-eosin (HE) staining for rat’s hearts. **(b)** TUNEL staining for rat’s hearts. **(c)** IL-6 concentration. **(d)** IL-8 concentration. **(e)** TNF-α concentration. **(f)** Western blots for GSK-3β/β-catenin signaling pathway.

To verify our findings *in vivo*, a rat I/R model was constructed. As it is illustrated in Fig. 5a, in the I/R treatment group, heart tissue appeared to be more swollen and disorganized, whereas the co-treatment of I/R and naringin reduced the inflammation. The results of TUNEL staining showed that naringin or miR-126 agomir treatment remarkably decreased the apoptosis of cardiomyocytes (Fig. 5b). Additionally, naringin or miR-126 agomir treatment reduced the mRNA expression levels of IL-6, IL-8, and TNF-α (Fig. 5 c-e), proving that naringin has anti-inflammatory effects on I/R. Next, we measured the activity of GSK-3β/β-catenin signaling in rat’s hearts. We discovered that naringin or miR-126 agomir decreased the phosphorylation of GSK-3β and promoted more β-catenin to enter nuclei in I/R rats (Fig. 5f). Thus, these data imply that I/R-induced heart damage and inflammation could be alleviated via miR-126/GSK-3β signaling pathway.

NS: non-significant; *P < 0.05; **P < 0.01; ***P < 0.001, *vs*. the control group.

## Discussion

This research has demonstrated that naringin could significantly reduce OGD/R-induced apoptosis of cardiomyocytes, as well as the release of inflammatory cytokines such as IL-6, IL-8, and TNF-α. Furthermore, our animal experiments proved that naringin shows protective impact on cardiac I/R injury. These results imply that naringin has the potential of improving the clinical outcome of patients with major cardiovascular diseases.

Initially, we have found that OGD/R treatment could remarkably impair the viability of cardiomyocytes, whereas naringin can restore their viability with its dosage increasing. In addition, the expression of cleaved caspase-3 and BAX can be downregulated by naringin, and the results of flow cytometry showed that naringin can decrease the apoptotic percentage of cardiomyocytes. To evaluate the inflammatory response of cardiomyocytes, the concentration of IL-6, IL-8, and TNF-α was measured, and we have discovered that the concentration of these cytokines induced by OGD/R can be reduced by naringin. These three cytokines have been shown to play a key role in the pathogenesis of cardiac I/R injury. Plasma IL-6 level is associated with worse clinical outcome of acute myocardial infarction^16^^,^ 17. In addition, there is a positive correlation between IL-8 level and heart failure^18^^,^ 19, and TNF-α can promote the apoptosis of cardiomyocytes^20^. Thus, our results showed that naringin can alleviate the apoptosis and inflammation of cardiomyocytes and has the therapeutic potential of cardiac I/R injury.

Furthermore, our research pointed out that naringin can exert cardioprotective effects via miR-126. Many researchers have reported that naringin can upregulate miR-126. Chen *et al*.^14^ found that naringin can increase miR-126 expression in lung carcinoma to mediate the anti-cancer effects of naringin. Also, Tzu-Wei Tan *et al*.^21^ demonstrated that naringin can suppress the migration of chondrosarcoma. It is worth mentioning that miR-126 has been acknowledged as an important miRNA that regulates cardiovascular diseases. It can be used as a biomarker for acute myocardial infarction and even for reducing the apoptosis of cardiomyocytes^22^^-^ 25. To confirm that miR-126 mediates the anti-apoptotic and anti-inflammatory effects of naringin, cardiomyocytes were transfected with miR-126 mimic or inhibitor. Upregulating miR-126 in cardiomyocytes that were exposed to OGD/R can have the same protective effects as naringin, while inhibition of miR-126 resulted in to abrogate the anti-inflammatory and anti-apoptotic effects of naringin in cardiomyocytes. In our animal experiments, upregulating miR-126 also reduces the cardiac I/R damage. Therefore, miR-126 is play key role to modulate the anti-inflammatory and anti-apoptotic effects of naringin.

In addition, we have discovered that miR-126 can target GSK-3β in cardiomyocytes, and this interaction is reported for the first time. Moreover, our *in-vivo* and *in-vitro* experiments have shown that naringin can promote the phosphorylation of GSK-3β and the relocation of β-catenin into nuclei. The pathophysiological role of GSK-3β has been widely studied. The phosphorylation of GSK-3β in cardiomyocytes can activate β-catenin. This can promote the proliferation of cardiomyocytes and maintain the survival of cardiomyocytes. The inhibition of GSK-3β has been shown to reduce cardiac I/R injury, modulate autophygocytosis and attenuate cardiac fibrosis^26^. It is of note that GSK-3β/β-catenin signaling can modulate the caspase-3-mediated apoptotic signaling pathway^27^. Moreover, GSK-3β inactivation can alleviate inflammation^28^^,^ 29. Considering these research findings and our results, naringin might regulate the apoptosis and inflammation of cardiomyocytes via modulating GSK-3β/β-catenin signaling.

However, our research has a few limitations. The first one is that more clinical data is required to verify the clinical significance of miR-126, and GSK-3β/β-catenin signaling in cardiac I/R injury. Second, clinical trials should be conducted to examine the efficacy of naringin in reducing cardiac I/R injury.

## Conclusion

Naringin can attenuate the apoptosis and inflammation of cardiomyocytes via miR-126/GSK-3β/β-catenin signaling pathway during cardiac I/R injury, which would provide a new insight into pharmaceutical treatment of cardiac I/R.
